# Horizontal cell connectivity in the anchovy retina—a 3D electron microscopic study

**DOI:** 10.1186/s12915-025-02242-7

**Published:** 2025-05-19

**Authors:** Petra Guder, Max Scheungrab, Peter Kohnert, Georgios Kolyfetis, Gerhard Wanner, Martin Heß

**Affiliations:** 1 Staatliches Museum Für Naturkunde Karlsruhe, Erbprinzenstraße 13, Karlsruhe, 76133 Germany; 2 Biozentrum LMU München, Großhaderner Straße 2, Planegg-Martinsried, 82152 Germany; 3 Zoologische Staatssammlung München, Münchhausenstraße 21, Munich, 81247 Germany; 4 Neurobiology University Konstanz, Universitätsstraße 10, Konstanz, 78464 Germany; 5 GeoBio Center LMU, Richard Wagner Straße 10, Munich, 80333 Germany

**Keywords:** Vertebrate retina, Polarization vision, Neural network, Volume electron microscopy

## Abstract

**Background:**

Block-face scanning electron microscopy has opened a new era of connectomics research, in which it is possible to make dense reconstructions of all cells in a clipping of a neuronal network, such as the retina, resolving synaptic contacts. Anchovies, exceptionally abundant marine teleosts, have retinae with regions for triple cone-based color vision and a region with specialized cone photoreceptors, so-called polycones, made of long and short cones with axially oriented outer segment lamellae for polarization contrast vision. This modality, discovered in the 1970s, is unique in vertebrates, but the neural wiring for contrast generation in deeper retinal layers is unknown so far.

**Results:**

To elucidate the retinal connectomics of the European anchovy *Engraulis encrasicolus* (Linnaeus, 1758), in a first project, we investigated the shapes and cone-specific wiring rules of 3 horizontal cell types using volume electron microscopy and subsequent computer-aided reconstruction: H1 cells contact both cone types of the polycone, H2 cells contact only the short cones, and H3 cells are exclusively connected to rods. In addition, a distinctive double band of Müller fibers and a layer of H1 axon terminals were structurally clarified.

**Conclusions:**

The findings suggest that (1) the monochromatic polarization contrast system based on fine structure specializations in the outer retina is connected to an inherited (bichromatic) color contrast mechanism in the inner retina, (2) the anchovy polycones arose from red (now long) and green (now short) cones, and (3) the blue single cones disappeared in the relevant retinal region.

**Supplementary Information:**

The online version contains supplementary material available at 10.1186/s12915-025-02242-7.

## Background

### Architecture and interspecific variations of the vertebrate retina

The eyes of vertebrates are important long-distance sensory organs used for orientation in the habitat, searching for food, avoiding enemies, finding reproductive partners or conspecifics in the swarm, and, last but not least, conveying people’s worldview. A central component is the neuroretina, which is simultaneously both an area sensor and imaging processor for the first neuronal processing stages as a derivative of the diencephalon. The cytoelectric excitation pattern at the level of the photoreceptor terminals is primarily picked up by horizontal (HC) and bipolar (BC) cells and forwarded in radial and horizontal signal processing pathways. The complex architecture of the retina, which consists of numerous, small, transparent, finely branched neurons arranged in three horizons of perikarya and two wiring horizons (plexiform layers) with their cell type-specific connectivity rules, eventually provides the structural basis for visual performance such as visual acuity, light sensitivity, contrast, motion vision, or separation of photopic and scotopic systems.

Essentially it follows a uniform organizational scheme [1] but shows various species-specific variations in detail, as a result of functional adaptations to the requirements of the photic habitats and visually controlled behaviors [2]. A prime example of retinal adaptations is the highly diverse group of teleosts in terms of the range of photoreceptor types [3], their geometrically regular horizontal arrangement patterns [4] and density distributions [5], the interconnection of the photoreceptor cells with the retinal pigment epithelium [6], the local ratios of the photoreceptor cells to secondary neurons and ganglion cells [7], and finally the geometry and wiring rules of their dendritic fields and axon terminals. These differences may also occur over the course of ontogeny [8] and/or within an individual’s retina [9]. The cone patterns of the outer retina usually continue in layers further inside the retina, recognizable for example in the HCs [10], in the inner plexiform layer [11], or in the ganglion cell layer [12].

The diversification of photoreceptor morphotypes was accompanied by the evolution of opsins with different spectral maxima covering the wavelength spectrum of the environment and as a prerequisite to perceive spectral contrasts (appropriate wiring provided). Already in early gnathostomes 5 classes of visual opsins had evolved (SWS1 (UV), SWS2 (blue), RH2 (green) and LWS (yellow or “red”) for cones, followed by RH1 for rods) enabling tetrachromatic vision [13–15]. Spectral tuning only requires the exchange of a few amino acids and thus a change in the charge pattern of the retinal binding pocket: see, e.g., discussions in [16, 17] and illustration in [18]: Fig. 15. Especially in teleosts, further gene duplications and mutations in the amino acid sequence (especially RH2 genes) led to diverse species-specific sets of opsins that can be fully or partially expressed [19–23].

### Polarization vision and the anchovy retina

Clupeiformes, a basal clade of teleosts, are trichromatic (having lost the SWS2 opsin) with bichromatic double cones (RH2_1/RH2_2 or RH2/LWS) in rows or square patterns in combination with “violet” single cones (SWS1) [17, 24]. Anchovies branched off about 50 million years ago [25] and in the course of their diversification they established, in addition to scattered single cones, mainly triple cones and also long and short cones connected to polycone rows in certain retinal regions [9, 26, 27]. In the subfamily Engraulidinae, a unique outer retina architecture has evolved [28–30], see also [6]: in the polycones of the ventro-temporal retina neighboring photoreceptor cells have radially oriented outer segment lamellae. The resulting structural dichroism makes them potential polarization analyzers with orthogonally oriented e-vector sensitivity, interpreted as an alternative way to create contrast in turbid water with low color contrasts. According to the suggested evolutionary path [6], some morphogenetic effects had to be combined to develop dual-channel analyzers for high-resolution polarization contrast vision. Comparable with the polarization pattern of clear skies, there is a more or less horizontally oriented band of moderately polarized light under water [31, 32]. The light reflected on the surface of objects (e.g., fish scales, plankton clouds) is depolarized [33, 34] and thus should clearly appear against the background in polarization contrast. General evidence for the specific perception of polarized light in the northern anchovy (*Engraulis mordax*) has been provided electrophysiologically and through behavioral experiments [35, 36] and biophysically in the bay anchovy (*Anchoa mitchilli*) [37]. In this context, it may be worth mentioning that anchovies are one of the most numerous vertebrates on this planet, and therefore their specialized eyes are all but a rare curiosity.

Since the discovery in *Anchoa mitchilli* and *A. hepsetus*, structural studies of the outer retina of other anchovy species have been published ([38, 39]: *Engraulis encrasicolus*, [26, 35]: *E. mordax*, [40]: *E. japonicus*, [41]: *E. anchoita*, [42]: *Coilia nasus*) and the idea of evolutionary processes within Engraulidinae [6]. With respect to the HCs of the anchovy retina, first indications can be found in [43] and [29]. In a TEM study by Heß et al. [44], three layers of HCs are described, one of which (H3) is embedded in the so-called Müllerband, and a first attempt at a 3D circuit analysis was made, although this is far from clear circuit diagram clarification despite appropriate efforts. Further time-consuming approaches with serial sectioning transmission electron microscopy (ssTEM) and elastic registration were undertaken (own unpublished studies), but were not pursued further owing to the difficulties inherent in the method, the suboptimal results, and above all, owing to the recent availability of volume electron microscopy (vEM). Further insights into the structure of the retina of *E. encrasicolus* come from cell density maps and ratio mapping [9, 43], which also target the inner retina [7, 45]. First neuroanatomical images obtained via fluorescence microscopy [45] depict BCs, HCs with putative axon terminals (HATs), and Müller cells (MCs). A density map of ganglion cells in *E. japonicus* was published by Miyazaki and Kobayashi [46].

### Structural elucidation of the retina

Owing to its complexity and the abovementioned structural properties (especially in the plexiform layers), the structural elucidation of the retina is methodologically demanding and, depending on the methods used, provides different details with specific resolutions and contrasts (i.e., shape of cells, extension of dendritic fields, termination depths in plexiform layers, and synaptic connections). Individual cells are usually marked stochastically (Golgi impregnation, DiOlistic labeling) or specifically (microinjection), or subpopulations of retinal neurons are labeled in their entirety (antibody staining, transgenic GFP expressing cell lines) and then imaged with light or electron microscopy via 2D or 3D approaches. Prominent examples from the extensive body of publications include widefield light microscopy [47], widefield fluorescence microscopy [48, 49], laser scanning microscopy [50, 51], ssTEM [52, 53], and CLEM [54–57].

The invention of volume electron microscopy two decades ago, either serial block-face scanning electron microscopy [SBF-SEM: [58]] or the focused ion beam-based FIB-SEM (DualBeam 620 from FEI company in 1993) and their biological application (e.g., [59]), including the retina (e.g., [60, 61]), changed the game. This method, in combination with computer-aided 3D segmentation and rendering, brought the deciphering of small connectomes such as retinal circuits within the realm of feasibility, including all cells involved and without any cell type-specific staining (see, e.g., Additional file 1: interactive Fig. S1).

### Objective of the study

The photoreceptor-specific interconnection of the three HC types in the anchovy retina is still unknown. The exact structure of the HATs is also still unclear, as is the specific nature of the “ Müllerband.” In the present study, volume EM scans were therefore made from the ventro-temporal retina of the European anchovy, with a first attempt at the level of the outer plexiform layer (OPL) and HCs. We aimed to (1) obtain structural data from the VOI (volume of interest) at EM resolution (i.e., cell types with their specific fine structural components/characteristics, number per volume/area, ratios, neuroanatomy, course of the H1 axons, and synaptic connections of the H1, H2, and H3 dendrites) and (2) make functional interpretations on the basis of these new data. Finally, for understanding the polarization contrast vision of vertebrates in the case of anchovies in terms of function and evolution, determining how the 2 polarization channels are further processed is important.

## Results

### Overall organization

**Fig. 1:**
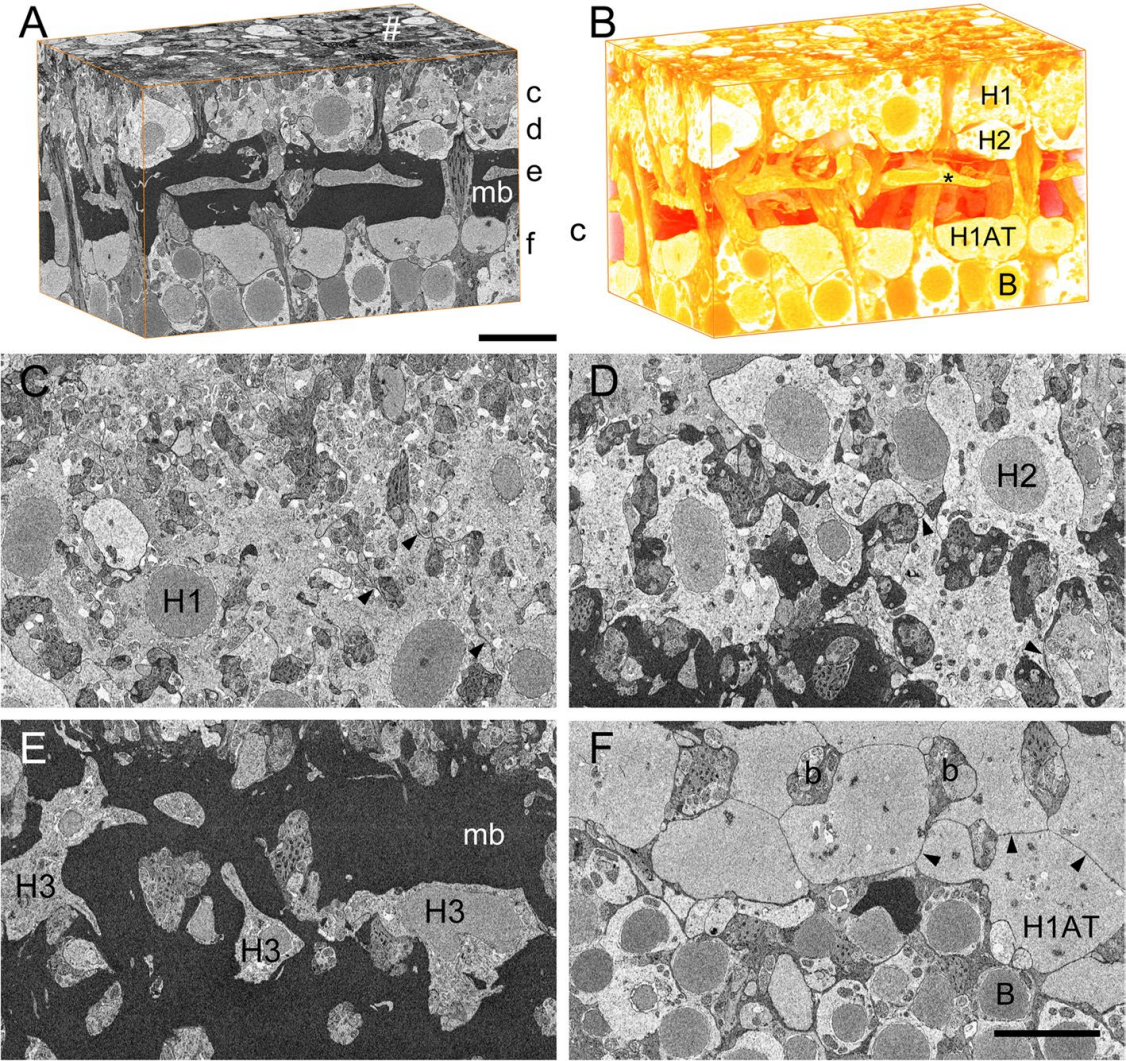
EM volume data from the ventro-temporal anchovy retina. **A** Cropped volume of Apreo scan 2 from horizontal cell dendrites (top) to scleral-most bipolar cell nuclei (bottom) represented by three orthogonal planes: XY front (cutting plane), YZ left, XZ top, inverted grayscale. Levels of digital reslices **C**–**F** indicated by c–f. **B** Volume rendering of **A**, highlighting less electron-dense structures, conveys the three-dimensionality of the structural data, especially at the level of the H3 cells. **C** XZ plane at the level of H1 cells. Nuclei surrounded by electron light cytoplasm. Between the lobed H1 profiles dendrite bundles of H2, H3, and BCs are seen. Arrowheads: neighboring H1 cells are connected by gap junctions. **D** XZ plane at the level of H2 cells. The space between the lobed H2 profiles is filled by dendrite bundles of H3 and BCs. Arrowheads: neighboring H2 cells are connected by gap junctions. **E** XZ plane at the level of H3 cells. **F** XZ plane (slightly oblique) at the level of horizontal cell axon terminals and bipolar nuclei. Arrowheads: neighboring H1 ATs are connected by gap junctions. # cone pedicles, B bipolar cell nucleus, b dendrite bundles of BCs, H1/2/3 horizontal cell type 1/2/3, * horizontal cell type 3, H1 AT horizontal cell type 1 axon terminal, mb Müllerband. Scale bars: **A** 10 µm (also **B**); **F** 10 µm (also **C**–**E**)

**Fig. 2:**
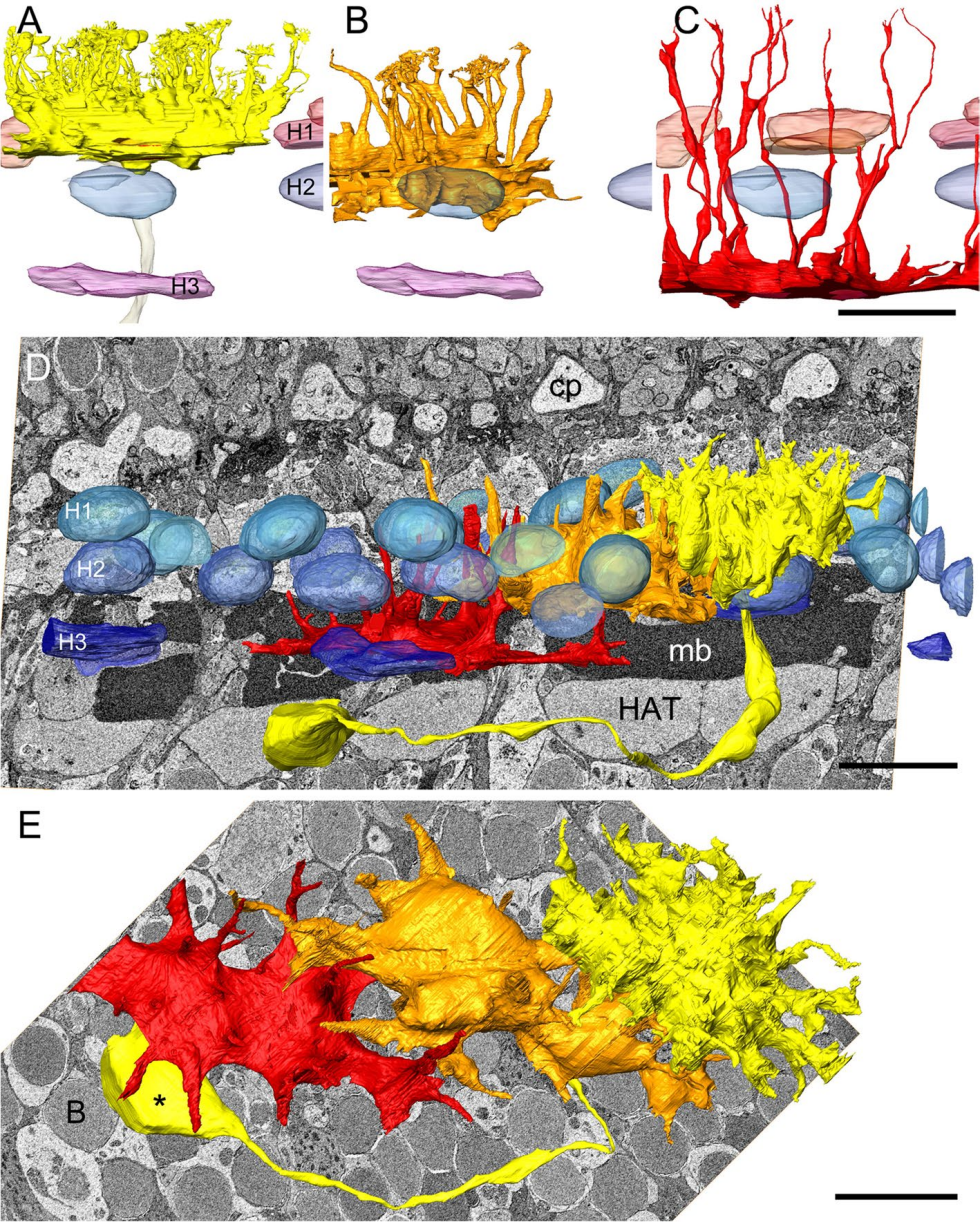
Layering and morphology of horizontal cells. **A**–**C** Surface renderings of horizontal cell somata and nuclei in radial view from FIB scan 5, **D** and **E** from Apreo scan 1. **A** Horizontal cell type 1 (yellow), H1–H3 horizontal cell nuclei. **B** Horizontal cell type 2 (H2, orange), H1 nuclei omitted. **C** Horizontal cell type 3 (H3, orange), H3 nucleus obscured. **D** Radial view of H1 cell with axon and axon terminal (yellow), H2 cell (orange), H3 cell (red), and H cell nuclei (H1–H3) in shades of blue. cp cone pedicle, HAT horizontal cell (type 1) axon terminal, mb Müllerband. **E** Horizontal view of a H1, H2, and H3 cell, nuclei not shown, * H1 axon terminal, B bipolar cell nucleus. Scale bars: **C** 10 µm (also **A**, **B**); **D**, **E** 10 µm

**Fig. 3:**
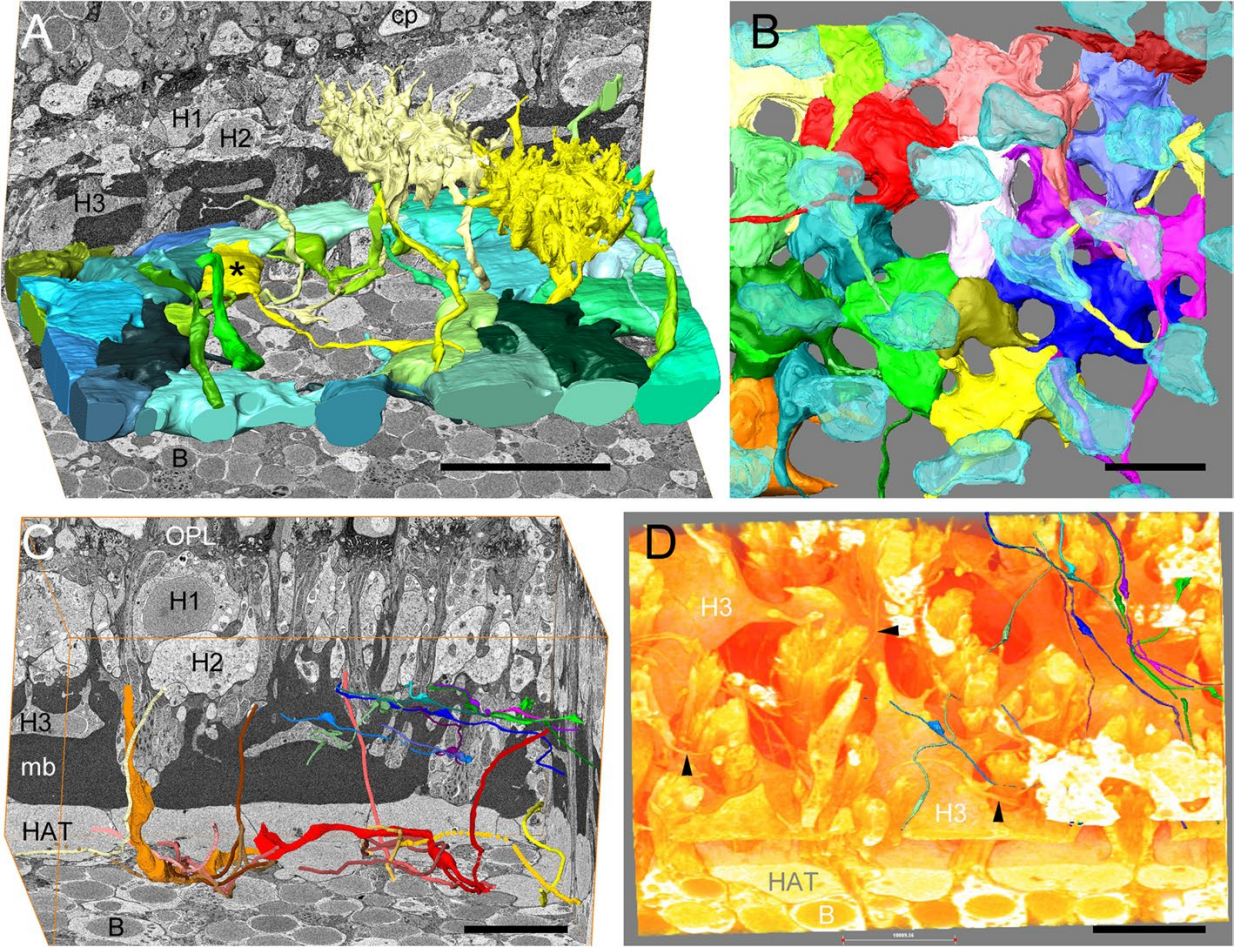
H1 axons with axon terminals, H2 axons. **A** Oblique view of the H1 axon terminal layer (in blue and green colors, by chance with a central hole formed by slightly displaced b-cells) with two reconstructed H1 cell somata (Apreo scan 1). * Axon terminal of yellow soma (see also Fig. 2D, E), B bipolar cell nucleus, cp cone pedicle, H1/2/3 horizontal cell type 1/2/3. **B** Horizontal view of the dense H1 axon terminal layer (with tiny holes for bipolar dendrite bunches) overlayed by H1 nuclei (semitransparent blue), ¼ of the stitched area of scan 4b, i.e., 1 tile of 4. **C** Apreo scan 2. H1 axons in warm colors (yellow to red, two completely reconstructed, the rest as skeletons, five reaching the H1 somata) and H2 axons in cold colors (green, blue, pink; skeletons with indicated varicosities). Labels as in **A** + HAT horizontal cell (type 1) axon terminal, OPL outer plexiform layer. **D** Volume rendering of the same data clipped by an oblique cutting plane just below the H2 somata showing H2 axons (arrowheads), partly as surface renderings. B bipolar cell, H3 horizontal cell type 3, HAT horizontal cell (type 1) axon terminal. Scale bars: **A** 20 µm; **B**–**D** 10 µm

**Fig. 4:**
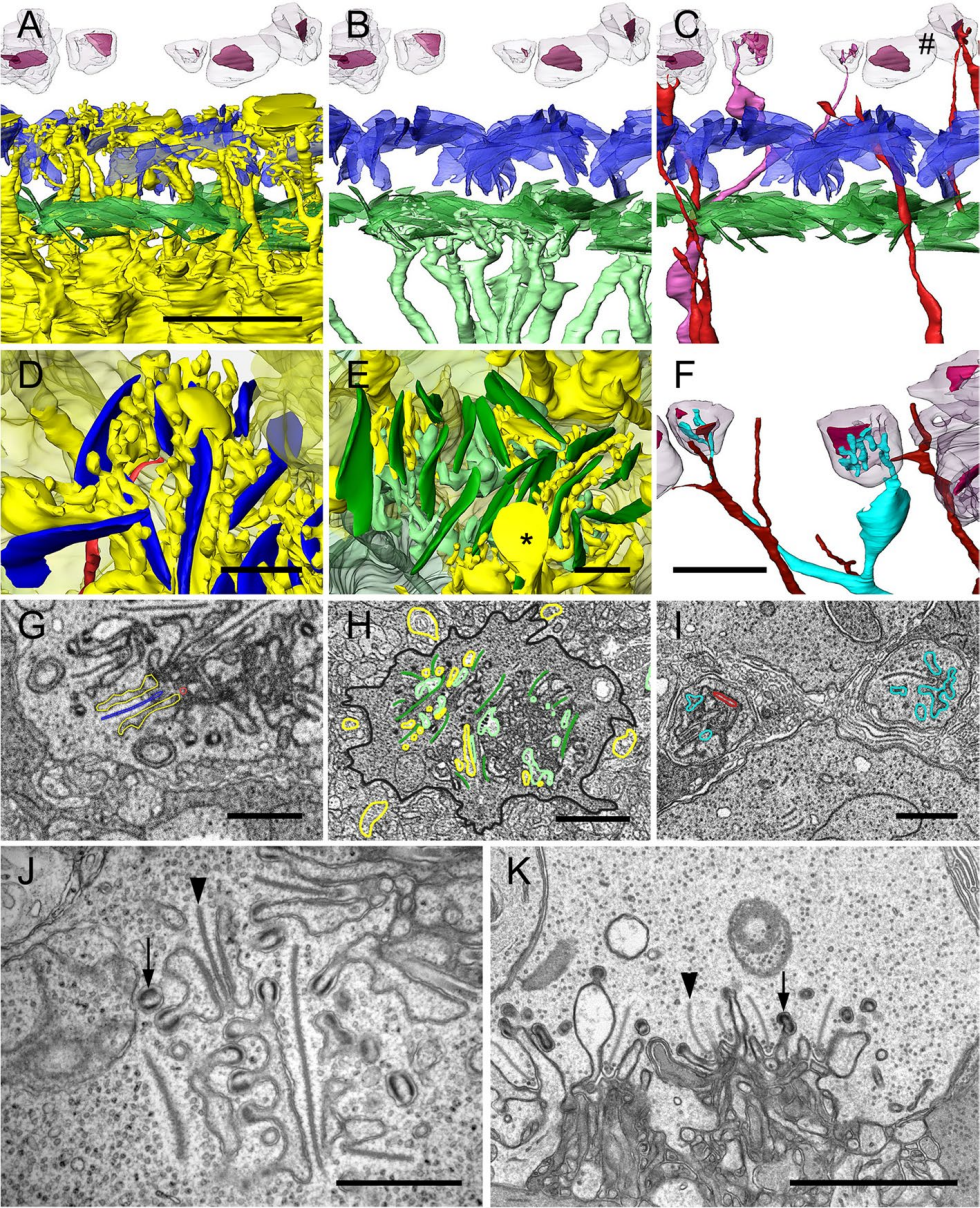
OPL connectivity of horizontal cells. **A**–**F** surface renderings from FIB scan 5, **G**–**I** segmentations from FIB scan 5, **J** + **K** STEM images. **A** H1 cell dendrites (yellow) contacting both, ribbons of short cone pedicles (green) and long cone pedicles (blue). **B** H2 cell dendrites (turquoise) contacting the ribbons of short cone pedicles (green) only. **C** H3 cell dendrites (red, pink) contacting the ribbons of rod spherules (#). **D** H1 dendrites (yellow) at the level of long cone ribbons (blue) and a bipolar dendrite. **E** H1 dendrites (yellow, with some artificial swellings *) and H2 dendrites (turquoise) at the level of short cone ribbons (green). **F** H3 dendrites (red, blue) at the level of rod spherules. **G** Selected EM plane showing the synaptic triad from **D**. **H** EM plane from **E**. **I** EM plane from **F**. **J** H cell dendrites with synaptic spinules invaginating a long cone pedicle (horizontal plane). **K** Radial plane showing two bunches of synaptic invaginations of a long cone pedicle. Arrows: spinules with postsynaptic densities, arrowheads: ribbons. Scale bars: **A** 5 µm (also **B**, **C**); **D**, **E**, **G**–**J** 1 µm; **F**, **K** 2 µm

The region of interest investigated in this study comprises the radial extension of the ventro-temporal retina of *E. encrasicolus* from the photoreceptor terminals to the scleral border of the BC layer (Fig. 1A, B). From scleral to vitreal, the following layers can be described: approximately ovoid rod spherules (Fig. 4A–C, F, I) fill the space between the vitreal-most rod perikarya and the long cone pedicles and contain a single synaptic ribbon each. The cone pedicles are pyramidal in shape (Figs. 2D, 3A) with a rectangular basal profile and multiple basal invaginations from second-order neuron dendrites and cone telodendrites (Fig. 4G, H, J, K). Occasionally terminal swellings of HC dendrites can be observed inside long cone pedicles, which are considered preparation artifacts (Fig. 4E). The slightly more electron-dense pedicles of short cones have bases a few µm vitreal to those of the long cones, both of which contain 16–18 synaptic ribbons and form a tessellated pattern (see [44]). Using scan 4a, the continuity of the two cone types of the polycone from the outer segments down to the two pedicle horizons was confirmed in this study (not shown). The photoreceptor density in the scans 1–3, with ca. 300 cones/10,000 µm^2^ (e.g., 118/3885 µm^2^ in scan 1), indicates a position of the examined retina fragments slightly dorsal to the area ventro-temporalis, the place of potentially sharpest photopic vision (see [9]: Fig. 7 A, [7]: Fig. 4A, [45]: Fig. 4D). Scan 4b with 450 cones/10,000 µm^2^ and 63 H1/10,000 µm^2^ is closer to the area ventro-temporalis than scan 1.

The thin, radially or slightly obliquely oriented dendrites of the horizontal and bipolar cells form the thin neuropil of the OPL, which is bordered vitreally by the somata of the H1 cells. The nomenclature of the HCs in the anchovy retina from H1 (scleral) to H3 (vitreal) follows Heß et al. [44]. The H1 and H2 cells lie in separate layers with touching somata (but no gap junctions between H1 and H2), and the planes of the nuclear centers of H1 and H2 cells are quite clearly defined and have a radial distance of ca. 4.5–5 µm in the examined retina fragments (Fig. 2A, D).

A striking structure is the “ Müllerband” vitreal of the H2 layer made of densely packed fibrous or rather tubular electron-dense material (Additional file 2: Fig. S2D; diameter of the tubules 25–30 nm determined from own unpublished TEM data), which is approximately 10–12 µm thick and houses the flattened H3 cells right in the middle. Between the Müller fibers, there are irregularly shaped sections of the MC cytoplasm with endoplasmic reticulum and sections of various secondary neuron dendrites. The connection with the MC somata, which run predominantly radially between the outer and inner limiting membrane, can best be seen at the borders to the BC dendrites. The “non-turgescent” MC with a rather electron-dense cytoplasm in the FIB stacks fill the narrow gaps between the neurons (partially not thicker than 70 nm, especially in the inner nuclear layer at the level of BCs and amacrines: Additional file 2: Fig. S2 A).

Between the vitreal border of the Müllerband and the scleral border of the BC somata, there is a distinctive, approximately 5–7.5 µm thick layer of less electron-dense, irregularly shaped, flattened “bubbles” (Fig. 1A, F), which turned out to be HATs (see below).

Viewed again from vitreal to scleral, these horizontal layers are interrupted by round to oval passages, which ultimately results in the lobe-shaped outlines of the HCs from a horizontal perspective, which are initially filled with B dendrite bundles. These are then joined by H3 dendrites and finally H2 dendrites before they contact the PRC terminals together with the H1 dendrites. Interestingly the horizontal extensions of neighboring MC with the fibrous material, encircling the dendrite bundles (Additional file 2: Fig. S2E), somehow follow the pattern of the HATs. This can be observed comparing the volume renderings of the vitreal surface of the Müllerband and the scleral surface of the HAT layer (Additional file 2: Fig. S2B, C).

For the following detailed reconstructions and descriptions, it should be noted that owing to the limited spatial extent of the VOIs, individual cells are only completely recorded in the center, but cropped in the periphery. Conversely, parts of cells that are predominantly outside extend into the VOI.

### Horizontal cells

The description of the morphology and areal density of HCs is based primarily on scans 1 and 5 and the corresponding 3D reconstructions.

H1 cells are roughly rectangular to oval in shape in radial sections with heights of 7 to 10 µm and a relatively electron-lucent cytoplasm (Fig. 1A–C). In horizontal sections, they are lobed and form full-surface gap junctions at all points of contact with neighboring H1 cells (Fig. 1C). Figures 2 and 3A show the 3D shape of H1 cells with up to 40 radial dendrites, leading toward the cone pedicles and further branching. Approximately 40% have an average length of 1.3 µm, 60% measure 4.5 µm (see below: OPL connectivity). The reconstructions from scan 5 result in a density of 150–160 primary dendrites/1000 μm^2^ retina area, equivalent to ca. 45 dendrites per H1 cell. This cell type has a clearly visible axon leading vitread (see next paragraph). The volume of the yellow H1 cell in Figs. 2D, E and 3 A (scan 1) amounts to 1110 µm^3^ without axon (computed from the count of calibrated voxels assigned to this cell), in Fig. 2A (scan 5) to 950 µm^3^.

When determining the density of only a few structural units within the measurement area, especially if there are partly complete and partly cut structures, incremental fluctuations can occur. Therefore, the density of the H1 cells was determined quite accurately from the volumes of the nuclei as follows: 12 nuclei with an average volume of 146.7 µm^3^ (± 8 µm^3^) were completely contained in the VOI of scan 1 under an area of 3884.65 µm^2^, and additional 5 cropped nuclei with a total volume of 110 µm^3^ corresponding to 0.75 cell nuclei were added. The surface density is therefore 32.8 cells per 10,000 µm^2^.

In radial sections, H2 cells are flatter and less electron dense than the H1 cells (height 5 to 7 µm); in horizontal sections, H2 cells are also irregularly stellate with slightly longer lobes (Fig. 1D) and they also contact neighboring cells of the same type over the entire surface with gap junctions. The three-dimensional shape of the H2 cells can be seen in Fig. 2B, D, and E; the H2 cells send fewer dendrites into the OPL (ca. 20–25/cell, ca. 45–55 dendrites/1000 μm^2^), which are longer than those of the H1 cells due to the stratification (ca. 7.6 µm). The volume of the H2 cell shown in orange in Fig. 2D and E is 1103 µm^3^; in Fig. 2B it is 1120 µm^3^. In the VOI of scan 1, seven H2 nuclei are completely included and seven are cropped. Analogous to the procedure for the H1 nuclei, there are 8.5 cell nuclei with an average volume of 148.8 µm^3^ under an area of 3884.65 µm^2^ or 21.9/10,000 µm^2^. The combined density of H1 and H2 nuclei of 54.6/10,000 µm^2^ points to the same retinal location as estimated from cone density in scan 1 (see [7]: Fig. 4D).

H3 cells, in radial sections, appear amidst the packages of horizontal Müller fibers with heights of 1.5 to 4 µm and comparatively high electron density (Fig. 1A, E). Owing to their comparatively long and thin horizontal lobes, their profiles change significantly depending on the cutting plane. In the horizontal section (Fig. 1E), the star-shape can only be seen when scrolling through a few section planes. This can be better observed in the 3D reconstruction (Fig. 2C–E), but can also be recognized in the inverted volume rendering between the MC layers that then appear transparent (Fig. 1B). Thin, radially oriented dendrites extend in scleral direction (length ca. 24 µm). Their number per cell is approx. 15 (i.e., 20 dendrites/1000 μm^2^), a residual uncertainty results from slight cropping of the cell at the edges. The result of the cell volumetry should also be a little too low with 272 µm^3^ in Fig. 2D, E and 117 µm^3^ in Fig. 2C. The contacts to neighboring H3 cells show broad gap junctions. Two of the ca. five H3 nuclei under the surface of the VOI suggest an average volume of 157 µm^3^ and an areal density of max. 13/10,000 µm^2^. This approximately matches the expected value of 10/10,000 µm^2^ in the retina area examined ([7]: Fig. 4E).

In general, the cell density and the density of the primary dendrites decrease from H1 to H3, but the area of the dendritic fields increases.

### Axons and axon terminals

The description of the HC axons and axon terminals is based primarily on scans 1, 2, and 4. Details were checked on scans 3 and 5.

In meticulous 3D reconstruction of H1 cells, an axonal projection is found at the vitreal base of all cells completely contained in the VOI, which extends vitreally, passing the H2 and H3 cells and ending in a structurally distinctive layer above the most sclerally located bipolar cell somata in a swelling (axon terminal; Figs. 2D, E; 3 A). Due to the non-rectilinear radial course, moreover with longer curves vitreal to the Müllerband, and the limited VOI, axons often cannot be followed completely from the cell somata into the terminal layer or vice versa. Accordingly, the axon terminals usually do not lie directly beneath the associated cell. In their descending part, axons vary in thickness between 1.1 and 2 µm and can become very thin (e.g., 200 nm), especially in their horizontal course on top or between the axon terminals. One H1 axon measured in the examined retinal fragment has a 3D length of ca. 73 µm, another a length of 65 µm.

The electron-lucent fine structure of the axon terminals is similar to that of the H1 somata, with fewer mitochondria but with groups of electron-dense granules (“nematosomata,” Fig. 1A, F). The terminals vary in size between ca. 6.5 × 12 and 12 × 15 µm in horizontal view (Fig. 3A, B). Like the somata of the associated cells, they are in broad contact with one another via gap junctions (Fig. 1 A, F). Gaps in the axon terminal layer appear where bundles of bipolar dendrites ascending from further vitreal require space. The terminals usually form a closed layer of ca. 4.5–8 µm radial thickness (Fig. 3B), which can, however, be interrupted in spots where bipolar somata bulge somewhat sclerally (Fig. 3A). Scan 1 contains 8 complete (mean volume 898 µm^3^) and 10 partially imaged HATs. Volumetrically, the area density of the HATs was determined to be 13.4 terminals per 3884.65 µm^2^ or 34.5 per 10,000 µm^2^. This density is only slightly higher than the local H1 density of 32.7 in scan 1 (apparently no terminals are missing in the central recess; rather, they have shifted laterally to the side). The repeated counting of axon terminals and H1 nuclei over a larger area (scan 4b: 42/6589 µm^2^, equivalent to 64/10,000 µm^2^, partly shown in Fig. 3B) also revealed that the two values were largely the same.

Accordingly, no axons of this dimension are found in H2 cells, not even in scan 3 (not shown, data in repository), which is three times higher resolved in XY than scan 1, nor in FIB scan 5. What we find, however (e.g., in scan 2), are very fine neurites sometimes only 120 nm in diameter and therefore difficult to trace in their entirety, which are partly coiled, partly run straight along the vitreal membrane of the H2 somata and/or just below them between the Müller fibers and sometimes repeatedly form varicosities on the H2 somata (Fig. 3C, D). If one removes all the cutting planes above them from the stack, these neurites (putative H2 axons) also appear in the volume rendering (Fig. 3D). The origin from a H2 cell was once determined. Synaptic structures, however, cannot be recognized given the data quality.

### OPL connectivity

For the digital disentanglement of the “dendrite tangle” of the OPL (Fig. 4G–K), the first substack of FIB scan 5 was used with very good contrast and high, almost isotropic resolution in all three spatial dimensions. The findings were verified via FIB scan 6 with similar resolution (data in repository). In the field-of-view (28 µm × 21 µm) of the horizontally sectioned scan 5, there were 14 short cone pedicles, 14 long cone pedicles, 3 of which were included completely without cropping each, and 25 rod spherules (for 14 of them, the contact with second-order neurons was tracked vitread). In a time-consuming manual segmentation step, all dendrite profiles within the field-of-view were traced individually from their synaptic contacts in the PRC to the cells of origin, i.e., H1–H3 dendrites and bipolar cell dendrites (the latter are not subject of the present study), complicated by invaginations of photoreceptor cell telodendrites. Occasional swellings at the tips of the HC dendrites (* in Fig. 4E) are considered fixation artifacts. This reconstruction revealed the expansion of the dendritic fields and cell type-specific wiring rules.

H1 dendrites contact the pedicles of both long cones and short cones (Fig. 4A, D, E, G, H) with a numerical preference for long cones (60%); H2 dendrites contact only short cones (Fig. 4B, E, H) and H3 dendrites contact only rod spherules (Fig. 4C, F, I). The dendrites of all HC types cling to the synaptic ribbons of the photoreceptor cells laterally and form numerous knobby “spinules” (diameter ca. 160 nm, Fig. 4D, J, K). This phenomenon is most noticeable in H1 dendrites. On the inside of the membrane of the spinules, there are approximately 20 nm thick electron-dense layers in the sense of postsynaptic densities. Each ribbon of a pedicle is therefore flanked on both sides over part or its entire length by the knobbed HC dendrites. In the case of the long cone ribbons, these are usually the dendrites of the underlying H1 cell on both sides, but on the periphery of the dendritic field the dendrites of two H1 cells can dock onto a ribbon opposite or next to each other—up to 4 cells can contact a pedicle. The same applies to the H1 and H2 contacts of the short cone ribbons, whereby the contact zone of the H2 dendrites is slightly more vitreal than that of the H1 dendrites. The dendritic fields of the H1 cells extended over at least 10 long cones and 8 short cones of three neighboring polycones in the examined VOI. Neighboring H1 cells show slightly overlapping dendritic fields at their periphery. H2 cells contact up to 7 short cones in the sample volume, but again up to three H2 cells can control the same pedicle. For the rod spherules, contacts of one or two H3 dendrites (also from different cells) were found.

## Discussion

### New data

With the 6 SBF-SEM scans created in the present study, perfectly aligned and practically gap-free volume data from the OPL and upper inner nuclear layer of the ventro-temporal anchovy retina are available in EM-typical resolution and contrast. This means that (1) any image plane can be selected to view cellular and subcellular components (including digital reslices), (2) volume renderings can be created to facilitate a 3D understanding of certain morphological aspects (see, e.g., Fig. 3D), and (3) “densely reconstructed” surface renderings can be worked out to represent the realistic shape of neurons and their cell type-specific circuitry, without any a priori cell labeling. Shrinkage artifacts due to chemical fixation are considered negligible here. Occasionally there were difficulties in tracking the finest branches in the dense tangle of unmarked fibers in the grayscale images when: (1) the system went out of focus for a short time, (2) very thin neurites ran parallel to the cutting plane, and (3) cell parts ran out of the sample volume or came in from outside.

Even if the final overall reconstruction with all reconstructed cells shown at the same time is still confusingly complex (like the view of a 2D section through a neuropil before), the particular strength of the method rests on the free combinability of the components in the digital surface models, the free choice of perspective, zoom factor, color, and transparency (Additional file 1: Fig. S1). This makes it possible to analyze (in the literal sense) and understand the intricately interwoven retinal nerve network, and to determine numerical ratios and volumes. In this case, it was possible to significantly expand the findings of Heß et al. [44] to depict the shape of the three HC types up to the tips of the dendrites completely and with high precision, and to determine their cell type-specific interconnectivity with different photoreceptor types in the OPL. The nature of the H1 axons with their terminals and the nature of the “ Müllerband” have now become clear.

### Layering and shape of H cells

Horizontal cells are found in the basic plan of the vertebrate retina at the scleral edge of the inner nuclear layer. In contrast to mammals [62, 63], in many groups of teleosts (and also in Chondrichthyes), a layer separated from the rest of the inner nuclear layer and consisting of two or more levels of HC nuclei is visible under the light microscope [64]. In goldfish [65], zebrafish [49], and carp [66], there are one or two H cell horizons, three in centropomids [67], whereas 4 layers can be distinguished among representatives of the mugilids, gerreids, lutjanids, and moronids [67–71]. The stratification of different subpopulations of H cells is commonly interpreted as a sign of different wiring and physiology, and thus as a sign of separate channels for the parallel processing of photoreceptor signals [65, 72], which can subsequently converge again for spectral contrast formation.

Initial descriptions of the HCs in anchovy retinae are brief and refer primarily to cell nuclei in light microscopic radial sections (or low-resolution TEM images): O’Connell [43] shows some H cell counts and nucleus diameters in *Engraulis mordax* and *Anchoa compressa*, and Fineran and Nicol [29] write about a “particularly well-developed horizontal cell layer” in *Anchoa mitchilli* and *A. hepsetus*. Heß et al. [44] described the layering of H1 to H3 cells and their shape in horizontal projection for *Engraulis encrasicolus* in more detail for the first time, also using a simple 3D approach. Koch et al. [7] analyzed the density distribution across the retina and differentiated H1 + 2 grouped from H3 via fluorescence microscopy.

A general functional interpretation of layering and cell shape is that in anchovies there are three HC types with different functions. From [45], we know that the calbindin concentration in HCs decreases from scleral to vitreal, which may indicate differences in their physiology. The spatial (and physiological?) isolation of the H3 somata by the Müllerband, which is strongly developed on both sides, also points toward functional differences or a special separation of the photopic and scotopic systems (see OPL connectivity below). The retinomotor function of rods vs. cones, which, in this regard, is typical in teleosts has been described also for anchovies [29]. H1 and H2 cells, on the other hand, show no structural isolation, even though they touch each other over a wide area—but this also applies to many other types of retinal neurons. There is an indication that the layering plays its role particularly in the area ventro-temporalis, the location of the highest cone densities ([45]: “in other parts of the retina this separation was less distinct or absent and the nuclei are larger”). Interestingly, the density distributions of the HCs (including the rod-related H3) and also of the MCs follow that of the cones ([7]: Fig. 4A, D, E; [45]: Fig. 3D–F, I), possibly due to a locally uniform proliferation process. This, however, due to the intense coupling of the individual levels via wide-area gap junctions as electrical syncytia, should not have a far too important meaning. There is also no pattern correlation between HCs and the course of the polycones.

### Müllerband

Since MCs fill the space between the somata of second-order neurons in a branched manner, they often appear blurred in light microscopic resolution (e.g., [73, 74]). A clear idea of the 3D structure of an MC in the vertebrate retina is presented, e.g., by Newman and Reichenbach ([75]: Fig. 1). For *E. encrasicolus*, a first highly resolved 3D model of an MC soma is shown in Additional file 2: Fig. S2 A of the present study, which basically resembles the vertebrate basic plan (e.g., [76])—except for the Müllerband in the scleral half of the HC layer. It appears to be linked with distinct HC layers and is also known from other genera within Engraulidinae ([6]: *Lycothrissa*, *Stolephorus*, *Encrasicholina*, *Lycengraulis*, *Anchoviella*). Other possible candidates, such as *Catostomus catostomus* [64] or *Roccus chrysops* [77], need electron microscopic reinvestigation.

### H cell axons and axon terminals

The evidence from fluorescence microscopy [45] concerning a structural and functional connection between the H1 layer and the previously not understood “bright” layer between the lower edge of the Müllerband and the upper edge of the BC nuclei was condensed to certainty by our 3D reconstructions of vEM data: H1 cells in the European anchovy form axons and a distinctive layer of axon terminals, H2 cells apparently form a fine network of axons at or close to their vitreal undersides, and H3 cells show no signs of axons in our analysis.

Axon-bearing HCs are common in the retinae of vertebrate species [78, 79] and also in other fish, either in all cone-contacting HCs [65, 80–82] or, as in anchovies, only in certain HC types ([49, 51]: zebrafish). Synaptic contacts of the axons can also be made with the perikarya of bipolars ([82]: goldfish) or with rods, as in higher vertebrates ([83]: tiger salamander; [84]: chicken; [52, 85]: primates).

The functional role of HC axons has been controversial in the past and has remained elusive or differs depending on the species under consideration. Ohtsuka [86] argues against the conductivity of HATs; other authors argue for active electric communication between the soma and HATs [87–89]. For the anchovy, this question can only be discussed in partial aspects, especially for the H1: (1) axon diameter and length certainly lead to conduction resistance, which, however, should not prevent the function of cytoelectric signal transmission. The transmitted signal should follow that of the H1 cell(s) and probably arrive at the HATs with a slight time delay. (2) The location of the HAT layer is considerably far from the cells of origin, and the formation of a well-developed electrical syncytium suggests an “independent” subtask in retinal signal processing. Sustained hyperpolarization may be maintained here and transmitted laterally (“sustained antagonistic surround” sensu [90]), parallel to the signal transmission on the H1 soma level. (3) Their location close to the BCs is likely to be functionally irrelevant, since there are no signs of synaptic connections with the BCs. (4) Nelson et al. [91] propose a “nutritive link.” Similarly, the presence of nematosomes could give the HAT layer the role of a metabolic reservoir. A reciprocal relationship between nematosomes and the postsynaptic densities of HC synaptic spines during light and dark adaptation is known (e.g., [92]).

### OPL connectivity

**Fig. 5:**
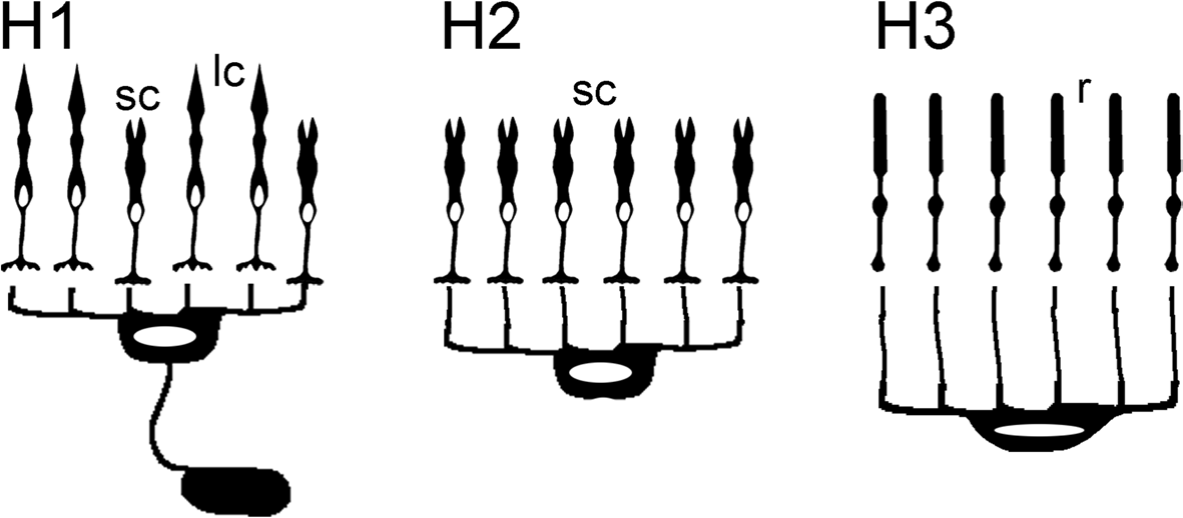
Schematic drawing of HC connectivity in the anchovy retina. H1, H2, H3 tree types of horizontal cells. lc long cones, sc short cones, r rods

A first, still imprecise image of H1 dendrites, which appear to run past the short cone pedicles and contact the long cone pedicles, is provided by Heß et al. ([44], Fig. 5c, d)—the calretinin-stained fluorescence image (Fig. 1G in [45]) already suggests that the H1 cells could have two termination horizons in the OPL. The assignment of H dendrites to cone patterns is, in principle, also possible via light microscopy and has been shown, e.g., in zebrafish [50, 93]. However, clear images of the HC-photoreceptor cell circuit with synaptic resolution have been obtained previously via TEM, usually from selectively contrasted cells on selected ultrathin sections or short ultrathin section series (e.g., [94]: *Carassius auratus*; [95]: *Rutilus rutilus*; [96, 97]: *Cyprinus carpio*). Connectomic analyses of the retina based on volume microscopy have been carried out by Behrens et al. on BCs [60] and HCs in the mouse [61]. The findings of the present work clearly show the HC-photoreceptor cell circuit in the anchovy retina: H1 cells actually contact long cone and short cone pedicles, H2 cells are selective for short cones, and H3 cells are selective for rods (Fig. 5). With this wiring selectivity, the picture of a functional separation of HC pathways for their inhibitory lateral feedback supporting contrast enhancement via cone-type opponent mechanisms (e.g., [98]) becomes more evident.

The basic HC connectivity pattern of the anchovy corresponds to that of other teleosts: the sclerally located H cells contact cones, and the vitreal H cell layer contacts the rods [51, 65, 67, 69, 70, 99]. In the goldfish, which has three cone-specific HC types, H1 cells form contacts primarily with red cones, but also with green and blue cones, H2 cells prefer green cones but also contact blue cones, and the H3 cells contact exclusively the blue cones [100]—the other way round, red cones only have contact with one HC type (H1), green cones with two HC types, and blue cones with three of them.

It is therefore possible to correlate the HC-photoreceptor cell connectivity pattern in the polycone regions of the anchovy retina with that of color-seeing fish and formulate the assumption that polarization contrast vision evolved from the color vision system: H1 and H2 correspond with the terminology of the above authors, H3 was lost, and the “H3” of the anchovy sensu Heß et al. [44] is the rod HC type (W. Stell, personal communication). Therefore, three further statements can be made: (1) *E. encrasicolus* has “lost” the blue cones (single SWS1 cones) in large parts of its retina, (2) the long cones could have evolved from the red cones (or better: the longer wavelength partner of double cones), and (3) the short cones from the green cones (the shorter wavelength partner of double cones). These statements are evident from the number of cone types and HC types in the ventro-temporal retina, from the wiring (longest wavelength cone connected with scleral-most HC type only, [100: Fig. 4]), and are also supported by current knowledge about the opsin types and spectra in anchovies: Since polarization contrast vision and color contrast vision are mutually exclusive as distinct modalities [101, 102], it is remarkable that the polycone components of the anchovy have the same spectral absorption maximum (λ_max_) rendering them monochromatic. Peak absorbance differs between anchovy species from 492 to 540 nm [16, 27, 36, 37, 103] corresponding to LWS and/or RH2 genes. Derived from the idea that they evolved from double cones [6], the opsins of long and short cones either converged or the expression ratio of opsin paralogs changed, or both. In the first case, only few amino acid substitutions at key positions would be sufficient to shift λ_max_ to match (for theory see, e.g., [104]). A second mechanism, based on the capability to express more than one opsin type per cone (see, e.g., [105–107]), seems no less plausible, possibly in combination with the former one: Kondrashev et al. [16, 27] demonstrated for another *Engraulis* species that the two members of polycones both express a single RH2 opsin with λ_max_ = 492 nm in the area ventro-temporalis, whereas two opsins (RH2: 492 nm and LWS: 512 nm) are coexpressed in the polycone regions outside the area with less regular outer segment lamellae orientation. Coexpression of both opsins results in a broadened peak at 502 nm with a content ratio of 50:50, but the latter can vary. In the “area” only, the shorter wavelength RH2 is expressed, guaranteeing monochromacy.

The evolution of color vision was an important step toward contrast formation and emancipation from overall brightness for better object recognition under difficult contrast conditions in water [108, 109]. Bichromatic color vision is supported by the dorsal and ventro-nasal retina of anchovies [36, 103] (see also [110]). The abandonment of the color system in regions of the retina that are directed forward and upward should have an advantage in the sense of the requirements for vision in murky water as described at the beginning. We assume that the specialization for high-resolution polarization contrast vision is limited to the cone outer segments and highly specialized pigment epithelium and is most likely based on the original, largely unmodified (now bichromatic) color contrast computer at the circuit level. Consequently, it was not necessary to “change a running system” and to invent a completely new contrast calculator with a new wiring scheme.

## Conclusions

The present study adds another successful example of determining wiring rules in a neural network via volume electron microscopy—an example that points to the open research field of retinal connectomics in the highly diverse taxon of teleost fish. Our findings suggest that the apomorphic e-vector-sensitive anchovy cones operate on a plesiomorphic, now bichromatic contrast mechanism. For this purpose, the original red and green cones would have been structurally changed and spectrally adjusted. The structure of the H1 axon terminal layer is now understood, but its function would have to be clarified physiologically (also in connection with the insulating layer of the Müllerband). The 3D EM datasets that were evaluated for HCs in this study contain extensive structural data on bipolar cells and (partially cropped at the edges of the VOI) amacrines, and ganglion cells. In a follow-up study, these are currently being evaluated for cell shapes, density values, and cell type-specific connectivity. In the future, other regions of the anchovy retina should be examined (e.g., the triple cone region, see [9]), as should the retinae of related species. Since segmentation for dense reconstructions of neuronal circuits is very time-consuming, user-friendly deep learning-based segmentation aids should be helpful in the future.

## Methods

To better understand the structural basis of polarization vision in anchovies, volume EM scans were created from the retina of the European anchovy and the cell shapes of HCs and their synaptic contacts with each other and with the photoreceptors were displayed via 3D surface renderings.

### Animals

European anchovies (*Engraulis encrasicolus* (Linnaeus, 1758), Engraulididae, Clupeiformes, Teleostei) were obtained from local fishermen on board their vessels (seine fishing with a light trap around new moons; Koper, Slovenia). From the ice-boxes, 6 animals of 12.5 cm standard length were selected; sex was not determined. Dead animals, ca. 1 h post mortem, were dissected and ca. 1 × 1 mm pieces of the ventro-temporal retina were fixed in 2.5% glutardialdehyde in 0.1 M cacodylate buffer + 3% sucrose overnight.

### Tissue preparation for SBF-SEM and creation of ApreoVS scans

While normal en bloc staining was sufficient for a good FIB-SEM contrast (see below), OTO staining modified after [111] was required for sample preparation for SBF-SEM: in brief, with intermittent washing steps, the prefixed retina samples were treated with 2% OsO_4_ + 1.5% potassium ferrocyanide on ice (90 min), thiocarbohydrazide solution at RT (20 min), 2% OsO_4_ at RT (30 min), 1% uranyl acetate overnight, and then 0.02 M lead aspartate at 60 °C (30 min). Following an ascending ethanol and acetone series, the samples were embedded in EMS hard plus resin 812, polymerized for 48 h at 60 °C, mounted on aluminum stubs with conductive glue (MG Chemicals 8331D) for radial or horizontal sections, and trimmed to 500 µm cubes with a diamond knife on an RMC MT-7000 ultramicrotome. A last semithin section from the block surface (0.5 µm thick, mounted on glass slides, and stained with Richardson’s reagent) was inspected under a light microscope for orientation. In addition, some EM images were obtained from ultrathin sections at 30 kV using an ApreoVS block face scanning electron microscope (Thermo Fisher Scientific Inc., Brno, Czech Republic) in STEM mode (Fig. 4J, K). Prior to 3D-EM investigation the mesa-preparations were sputtered with 20 nm gold using a Leica EM ACE 200 sputter coater with an integrated thickness monitor.

Four vEM scans were performed using the Apreo Volume Scope with inserted microtome with alternating diamond knife cutting and SEM scanning. The process was automated via the system’s own MAPS software with the following scanning parameters: high vacuum mode, working distance 6.5 mm, 1.78 kV, 0.1 nA, 1–3 µs pixel dwell time, and T1 backscattering electron detector.

Scan 1: radial orientation, 3080 px × 7352*px × 1009 planes @ 25 × 25 × 50 nm (8 bits, 22.8 GB), * from cone outer segments to the scleral border of the inner plexiform layer. For the evaluation of the HC layer, the stack was cropped in Y to display the region from the OPL to the most scleral BC nuclei (3080 px × 2025 px × 1009 planes =  > 77.0 µm × 50.625 µm × 50.450 µm, 6.4 GB) and the grayscale was inverted for a TEM-like impression of the image planes.

Scan 2: radial orientation, 5308 px × 3320* px × 677 planes @ 10 × 10 × 50 nm (16 bits, 23.9 GB), * from cone outer segments to the most scleral BC nuclei. For evaluation, the stack was converted to 8 bits, contrast enhanced and inverted (= > 53.08 µm × 33.2 µm × 33.85 µm, 13.5 GB).

Scan 3: radial orientation, 4504*px × 7126 px × 606 planes @ 8 × 8 × 40 nm (2 tiles with 15% overlap, stitched, 16 bits, 2 × 18.1 GB) =  > 36.03 µm × 57.01 µm × 24.25 µm, * from cone outer pedicles down to the level of H1 axon terminals.

Scan 4a: horizontal orientation, 6144 px × 6144 px × 92* planes @ 20 × 20 × 1000–3000 nm (8 bits, 3.23 GB) =  > area of 122.88 µm × 122.88 µm, * from cone outer segments down to the level of H1 nuclei.

Scan 4b: horizontal orientation, 2 × 2 tiles á 6144 px × 6144 px × 656*planes @ 8 × 8 × 40 nm (8 bits, 4 × 23 GB) with 20% overlap, then stitched and cropped to 10,147 px × 10,147 px × 656 planes =  > 81.18 µm × 81.18 µm × 26.24 µm, * from H1 nuclei to the scleral-most BC nuclei. For Fig. 3B, the scleral half (319 planes) of 1 tile containing the H1 ATs between the lower Müllerband and scleral-most B-cell nuclei was used.

### Tissue preparation for FIB-SEM and creation of Auriga scans

Small pieces from the ventro-temporal retina close to the area ventro-temporalis (containing polycones at high photoreceptor density = small cell diameters; see [9]: Fig. 7 A) were postfixed in 1% OsO_4_ solution in 0.1 M cacodylate buffer (2 h, 0 °C), en bloc stained with 4% uranyl acetate (1 h, 40 °C), dehydrated in a graded acetone series, and embedded in epoxy resin (Glycidether 100). After polymerization (2 days, 60 °C), the blocks were trimmed with a glass knife on an RMC MT-7000 ultramicrotome for radial slices, mounted on aluminum stubs with two-component conductive epoxy glue (ITW Chemtronics), and trimmed with a 90° diamond trimming knife to a mesa-shape (see, e.g., [112]: Fig. 4.18) with polycone rows oriented perfectly parallel to the upper surface. Laterally the photoreceptor layer was cropped away with glass knives until the outer OPL at the level of the rod terminals was just reached; the resulting vertical block face allowed (lateral) milling of the sample with the focused ion beam in horizontal planes in relation to the retina. The last semithin section from the upper block surface was inspected under the light microscope for overview. The samples were polymerized again (2 days, 100 °C) and carbon coated (15 nm) with a Balzers High Vacuum Evaporator BAE 121 + BalTec QSG 100 Quartz Film Thickness Monitor. Alternating focused ion beam milling and SEM imaging were performed on a Zeiss Auriga 60 dual beam workstation (Carl Zeiss Microscopy, Oberkochen, Germany) after the deposition of a 500-nm platinum layer on the mesa surface above the final VOI: 50 × 30 µm cutting window, 27.9 × 20.9 µm scanning window. The milling and imaging parameters were as follows: 500 pA milling current of the Ga-emitter, aperture size of 60 μm, high current mode at 1.5 kV, in-lens EsB detector with the EsB grid at − 1.4 kV, line averaging of 4 ×, and a pixel dwell time of 7 µs.

The FIB scan (scan 5) covered the retina from cone pedicles to H1 axon terminals (1501 horizontal planes, z-range 25.9 µm, 11.3 GB) in 2 substacks, once retrimming the block-front against shadowing effects in a deep milling notch. The resolution was adapted to the size of the relevant structures to reduce the scanning time, cost, and data volume: the OPL (cross section of finest bipolar cell dendrites and H cell spinule necks ca. 50–75 nm) was scanned with a voxel size of 9.07 × 9.07 × 10 nm^3^ (1229 planes with 3072 × 2304 pixels, field-of-view 27.9 × 20.9 µm^2^). In adaptation to the less fine structures vitreal of the OPL, the upper inner nuclear layer was imaged with a voxel size of 13.6 × 13.6 × 50 nm^3^ (272 planes á 2048 × 1536 pixels, field-of-view again 27.9 × 20.9 µm^2^).

A second FIB stack (scan 6) covered the OPL from rod spherules to scleral-most parts of H1 cells (973 consecutive horizontal planes á 3072 × 2304 pixels, voxel size 9.04 × 9.04 × 10 nm^3^, field-of-view 27.8 × 20.8 µm^2^, z-range 9.7 µm, data volume 6.43 GB).

### Digital imaging

Preprocessing (translational fine alignment, denoising, contrast optimization, cropping, and grayscale inversion) was performed with Fiji software [113]. Volume rendering, surface rendering, and selection of scenarios were performed with Amira 5.6 software (Thermo Fisher) on a Fujitsu Celsius R670-2 PC (32 GB RAM, Intel Xeon E5-1603 processor @ 2.8 GHz, Nvidia Quadro K2000 graphics card) using a Wacom interactive pen display DTF-720. All segmentations were performed manually.

## Supplementary Information

Additional file 1. Additional Fig. S1 Selected details of horizontal cell connectivity in the anchovy retina. A Clipping of the outer plexiform layercontaining two long cone pediclesand a short cone pedicleto illustrate the complex OPL membrane system and the quality of the raw data. B Data stack and magnification as in A with semitransparent superimposition of the structures shown in C and D. C Radial view of a H1 celland a H2 cellwith their dendrites contacting the synaptic ribbons of shortand long cones. D Horizontal view: the ribbon synapses of long cones are flanked only by H1 dendrites, and those of short conesare flanked by H1 and H2 dendrites

Additional file 2. Additional Fig. S2 Müller cells and the Müllerband. A Surface rendering of a Müller cell soma (only the part within the inner nuclear layer) in radial view “deformed” by the nuclei of bipolar and amacrine cells, two of the latter highlighted in pink. The scleral (top) and vitreal (bottom) processes are not followed to their ends at the outer and inner limiting membranes. Also, the horizontal spread scleral of the inner nuclear layer is not reconstructed. From FIB scan 5. B Volume rendering of the Müllerband shown from its vitreal side (note structure units “ + ” that correspond to the HATs “o” of C in shape), Apreo scan 2. C Volume rendering of the same stack (inverted!), cropped slightly below the scleral surface of the HATs showing their horizontal pattern. D Radial section of the Müllerband (* H3 cells), STEM. E Horizontal FIB image of the Müllerband (scleral half) with structural units surrounding bundles of BC + H3 dendrites: Bb. Arrowheads: Müller cell cytoplasm. H2 horizontal cell type 2. Scale bars: A 5 µm; C 10 µm (also B); D 2 µm; E 5 µm

## Data Availability

The datasets generated and/or analysed during the current study are available in the Electron Microscopy Public Image Archive (https://empiar.org/, [114]) under the accession codes EMPIAR-12585 (scan 1), EMPIAR-12586 (scan 2), EMPIAR-12587 (scan 3), EMPIAR-12591 (scan 4b), EMPIAR-12592 (scan 5 part 1), EMPIAR-12610 (scan 5 part 2), and EMPIAR-12609 (scan 6).

## Abbreviations

2D: Two dimensional
3D: Three dimensional
BC: Bipolar cell
CLEM: Correlative light electron microscopy
EM: Electron microscopy
FIB: Focused ion beam
GFP: Green fluorescent protein
λ_max_: Absorption maximum (wavelength)
H1–3: Horizontal cell types 1–3
HAT: Horizontal cell axon terminal
HC: Horizontal cell
MC: Müller cell
OPL: Outer plexiform layer
RT: Room temperature
SBF-SEM: Serial block-face scanning electron microscopy
SEM: Scanning electron microscopy
ssTEM: Serial sectioning transmission electron microscopy
STEM: Scanning transmission electron microscopy
TEM: Transmission electron microscopy
vEM: Volume electron microscopy
VOI: Volume of interest
