# Supplementary material for: Horizontal cell connectivity in the anchovy retina—a 3D electron microscopic study
Source: BMC Biol. 2025 May 19;23:137. doi: 10.1186/s12915-025-02242-7 (PMC12090589; doi:10.1186/s12915-025-02242-7)
Supplement: Supplementary file 1 — Additional file 1. Additional Fig. S1 Selected details of horizontal cell connectivity in the anchovy retina. A Clipping of the outer plexiform layercontaining two long cone pediclesand a short cone pedicleto illustrate the complex OPL membrane system and the quality of the raw data. B Data stack and magnification as in A with semitransparent superimposition of the structures shown in C and D. C Radial view of a H1 celland a H2 cellwith their dendrites contacting the synaptic ribbons of shortand long cones. D Horizontal view: the ribbon synapses of long cones are flanked only by H1 dendrites, and those of short conesare flanked by H1 and H2 dendrites [file 12915_2025_2242_MOESM1_ESM.pdf]

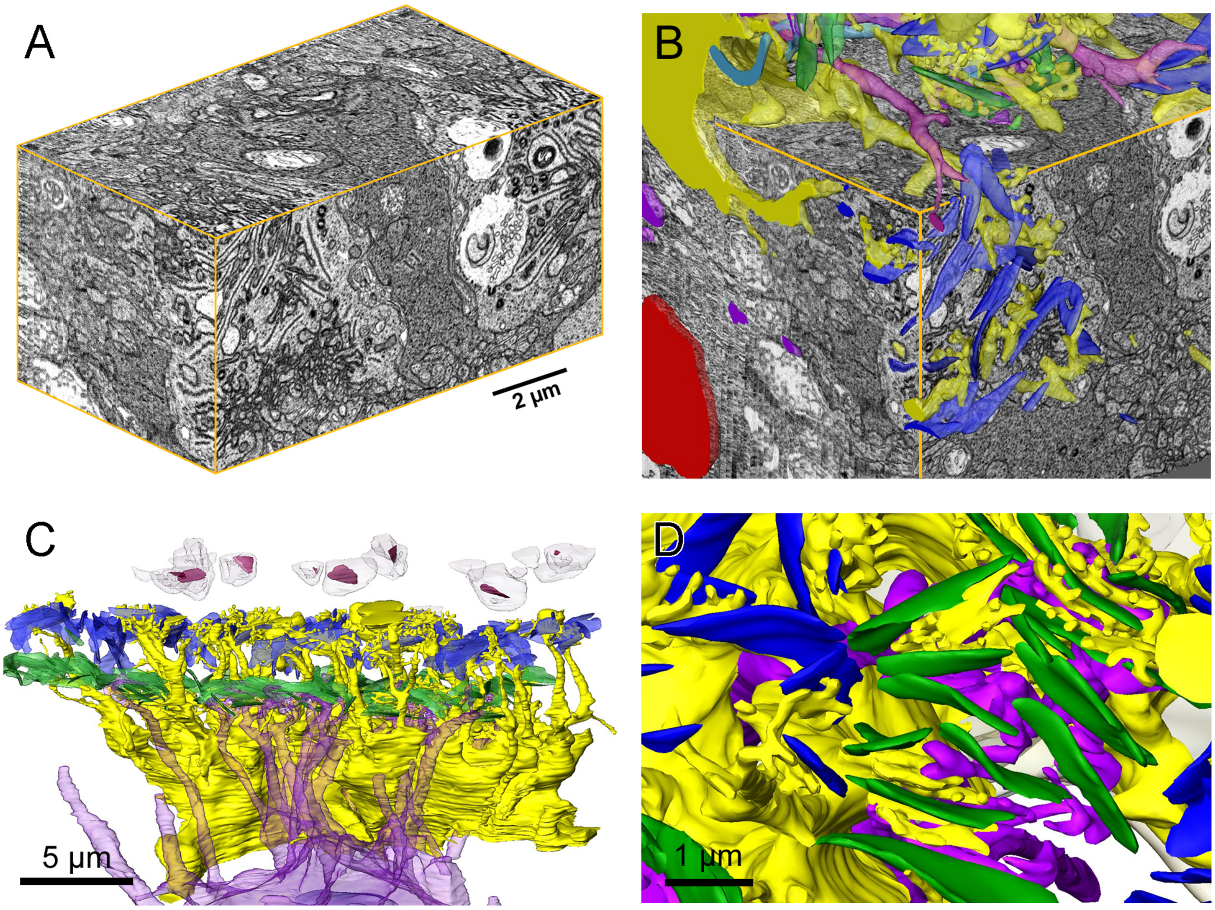

Supplementary Figure 1: Selected details of horizontal cell connectivity in the anchovy retina
A) Clipping of the outer plexiform layer (opl) containing two long-cone pedicles (left and right, electron-light cytoplasm) and a short-cone pedicle (center, electron-dense cytoplasm) to illustrate the complex opl membrane system and the quality of raw data (10 nm isotropic, perfectly aligned). B) Data stack and magnification as in A with semitransparent superimposition of the structures shown in C and D. C) Radial view on a H1-cell (yellow) and H2-cell (purple) with their dendrites contacting synaptic ribbons of short (green) and long cones (blue). The ribbon-synapses of long-cones are flanked only by H1-dendrites (note spinules), those of short-cones (terminating further vitral) by H1- and H2-dendrites. To access **interactive 3D-reconstructions** (Acrobat reader 7.0 or higher required) click into the figure. Switch between prefab views (central left window) or select components in the model tree. Rotate model by dragging with left mouse button pressed, shift model: same action + ctrl, zoom: use mouse wheel (or change default action for left mouse button).
