# Supplementary figures and images for: Horizontal cell connectivity in the anchovy retina—a 3D electron microscopic study
Source: BMC Biol. 2025 May 19;23:137. doi: 10.1186/s12915-025-02242-7 (PMC12090589; doi:10.1186/s12915-025-02242-7)

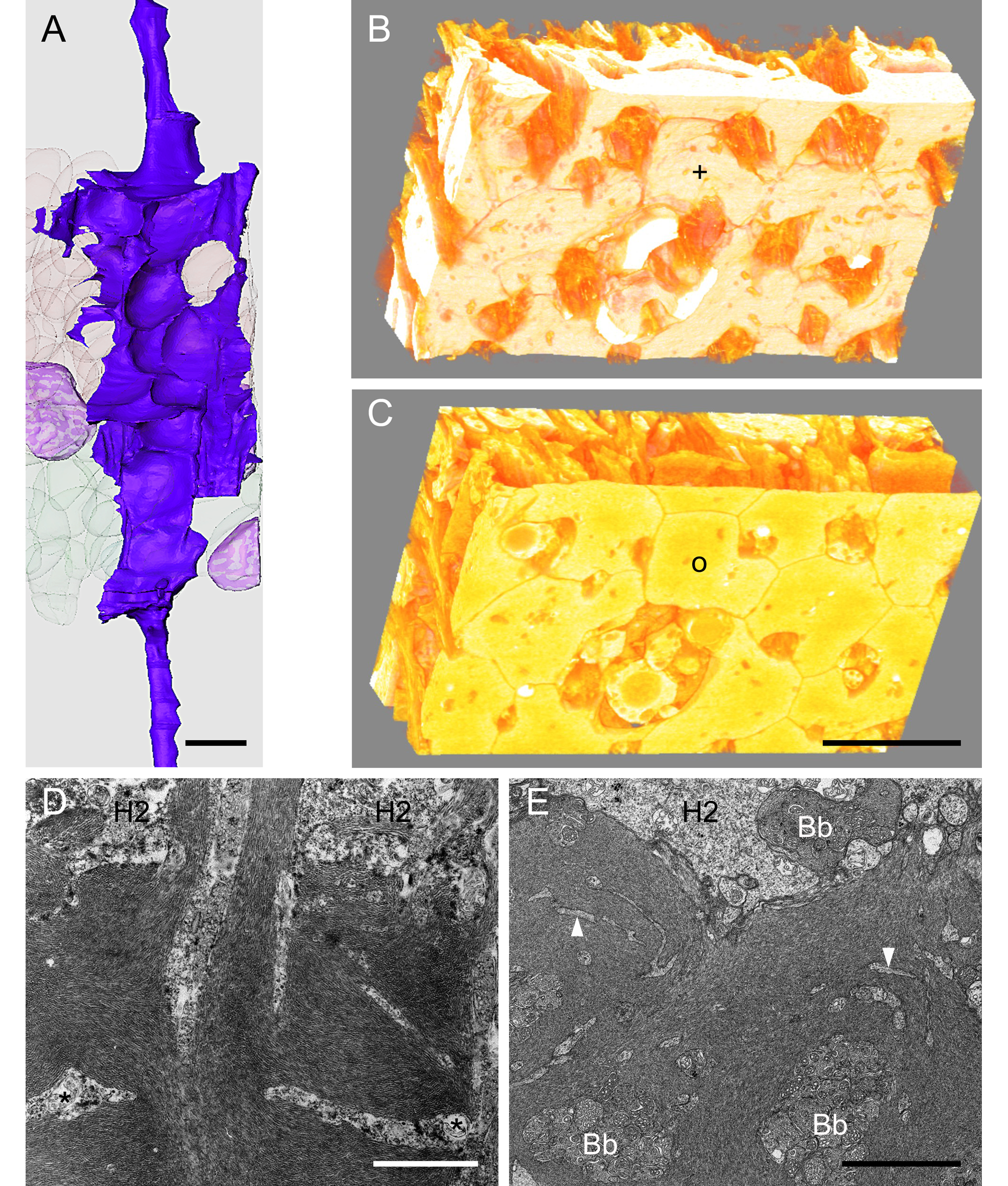

Supplement: Supplementary file 2 — Additional file 2. Additional Fig. S2 Müller cells and the Müllerband. A Surface rendering of a Müller cell soma (only the part within the inner nuclear layer) in radial view “deformed” by the nuclei of bipolar and amacrine cells, two of the latter highlighted in pink. The scleral (top) and vitreal (bottom) processes are not followed to their ends at the outer and inner limiting membranes. Also, the horizontal spread scleral of the inner nuclear layer is not reconstructed. From FIB scan 5. B Volume rendering of the Müllerband shown from its vitreal side (note structure units “ + ” that correspond to the HATs “o” of C in shape), Apreo scan 2. C Volume rendering of the same stack (inverted!), cropped slightly below the scleral surface of the HATs showing their horizontal pattern. D Radial section of the Müllerband (* H3 cells), STEM. E Horizontal FIB image of the Müllerband (scleral half) with structural units surrounding bundles of BC + H3 dendrites: Bb. Arrowheads: Müller cell cytoplasm. H2 horizontal cell type 2. Scale bars: A 5 µm; C 10 µm (also B); D 2 µm; E 5 µm [file 12915_2025_2242_MOESM2_ESM.tif]
